# Development of a General Fabrication Strategy for Carbonaceous Noble Metal Nanocomposites with Photothermal Property

**DOI:** 10.1186/s11671-019-3242-1

**Published:** 2020-01-21

**Authors:** Hongmei Zhu, Xuchuan Jiang

**Affiliations:** 1 School of Mechanical Engineering, University of South China, Hengyang, 421001 Hunan China; 2 Department of Chemical Engineering, Monash University, Clayton, VIC 3800 Australia

**Keywords:** Carbonaceous nanocomposite, Hollow carbonaceous structure, Hydrothermal synthesis, Etching process, Photothermal effect

## Abstract

**Figure:**
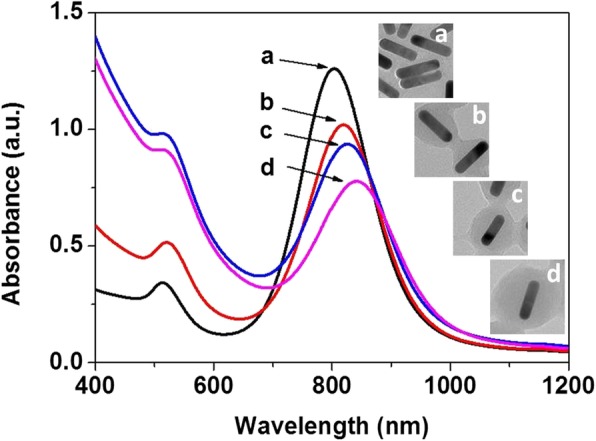
This study demonstrate a facile hydrothermal synthesis of noble metal carbonaceous nanocomposites (e.g., Au@C) with simple procedures under mild conditions, which can be25expanded as a general method for preparing diverse carbonaceous core-shell nanoparticles. The Au@C carbonaceous nanostructures exhibit interesting UV-Vis properties dependent upon shell thickness.

This study demonstrate a facile hydrothermal synthesis of noble metal carbonaceous nanocomposites (e.g., Au@C) with simple procedures under mild conditions, which can be25expanded as a general method for preparing diverse carbonaceous core-shell nanoparticles. The Au@C carbonaceous nanostructures exhibit interesting UV-Vis properties dependent upon shell thickness.

## Highlights

- Develop a simple hydrothermal method for generating noble metal@C core-shell nanostructures
- The generated Ag or Au cores are well crystallized, but the carbonaceous sheath is amorphous
- Glucose plays a multiple role as a reductant for metallic ions, a shape modifier and surface protection
- Low-temperature (60–100 °C) hydrothermal reaction for etching Ag@C core-shell to obtain hollow carbonaceous sheath
- Rodlike Au@C nanocomposites show photothermal effect, potential for biomedical applications

## Introduction

Carbon materials have become more and more important in material science and technology. A number of carbon particles have been investigated in the past, such as carbon nanotubes, carbon spheres/dots [1–3], and graphene oxide (GO) [4, 5]. Among them, carbonaceous nanostructures have exhibited some unique properties such as good stability (< 200 °C), porous tubular walls, superior lubricating properties, easily dispersing in water, and biocompatibility, as well as readily hybridized with metals (e.g., Au, Ag) and/or metal oxides for generating hybrid functional nanostructures [6–13].

Specifically, carbonaceous metal nanocomposites have attracted more interests due to its excellent biocompatibility in medical applications. To achieve such carbonacesous particles or their metallic hybrid structures, a number of methods have been developed, e.g., hydrothermal method, using sugar or starch as a starting material [14], for example, one-step hydrothermal synthesis of carbonaceous silver nanocables and nanotubes after etching silver core s[15], a template approach for preparing Ag/Cu@poly(vinyl alcohol) (PVA) coaxial nanocables, tellurium (Te)@PVA nanostructures [16], and porous membrane-based template and positive hard templates [17–20] for the synthesis of carbonaceous nanostructures. However, the removal of the hard template(s) may lead to problems in post-treatments (chemical etching or high-temperature calcinations) such as structure broken or collapse [14–22]. In addition, a little was reported to elucidate the formation/evolution mechanism of carbonaceous noble metal nanostructures achieved under the reported conditions.

Photothermal therapy has been developed because it has less damage to healthy tissues via generating localized heat especially in early metastasis steps or when the tumor is in the primary stage. Gold nanoparticles (GNPs) are one of the excellent selections for this approach. Recent advances in the multi-functional design of GNPs allow for the generation of localized heat in the proximity of cancer tissues and additionally allow the delivery of multiple desired drugs in a controlled and targeted manner. GNPs have many benefits that make them suitable for the photothermal treatment of tumor or cancer, because they can be delivered into the local tumor area while minimizing non-specific distribution, activated via near-infrared (NIR) laser light, creating the ability to penetrate deep into biological tissues, and modulated to create multifaceted cancer photothermal therapy [23–25].

Herein, we demonstrate a simple but effective synthesis method for generating carbonaceous Au@C or Ag@C nanostructures under mild conditions (180–200 °C). Interestingly, the Ag cores rather than Au ones can be etched or removed from the carbonaceous structure by low-temperature etching (60 °C for tens of hours) with no need of any high-temperature treatments. The microstructure of the as-prepared nanocomposites will be characterized, and the possible formation mechanisms will be understood. The photothermal property of Au@C nanostructures, as a case study, will be examined, by referring to recent studies [23–25]. This study may offer a facile but effective strategy to prepare carbonaceous metal nanocomposites with potential applications in material science, catalysis, and biochemistry.

## Experimental Method

### Chemicals

The following chemicals were purchased from Sigma-Aldrich and used as received without further purification: silver nitrate (> 99%), **d**-glucose (99%), cetyltrimethylammonium bromide (CTAB, > 98%), and gold (III) chloride trihydrate (HAuCl_4_·3H_2_O, > 99.9%). All glassware was cleaned with fresh aqua regia, rinsed extensively with distilled water and/or ethanol for a few times, and dried before use.

### Synthesis of Carbonaceous Ag@C and Au@C Nanostructures

Typically, a few steps were involved in the synthesis procedure. **In s**tep 1, 2.0 mL 0.01 M of AgNO_3_ and 6.0 mL 0.01 M glucose solution were added into a 50-mL glass beaker containing 10 mL of 0.12 M CTAB solution, then mixed and stirred to make sure homogen**eit**y. **In s**tep 2, the total volume of the mixture solution was fixed at 35 mL using distilled water under stirring over 10 min for homogeneity. The color of the mixture solution gradually became light-yellow color, probably due to the formation of AgBr precipitate in the presence of CTAB. And **in** step 3, the mixed solution was transferred into a stainless steel autoclave with a Teflon liner of 50-mL capacity and heated in an oven at 180 **°**C for a couple of hours.

Similar **to the** procedures to prepare Au@C nanoparticles, the replacement of AgNO_3_ by HAuCl_4_·3H_2_O solution was conducted in this work. In terms of the preparation of carbonaceous Au@C nanorods, the Au nanorods were synthesized first, based on our previous work [26**–**28], in which the CTAB plays a key role in controlling **the** formation of Au nanorods. The Au nanorods were dispersed in glucose solution for further hydrothermal reaction at 180 °C for 6**–**24 h.

### Etching for Hollow Carbonaceous Structure

Hollow carbonaceous nanostructures were fabricated by etching Ag cores from Ag@C nanocomposites by keeping the same reaction solution at 60 **°**C for tens of hours, while this is not workable for etching Au core from Au@C. Through a dynamic reaction process, the Ag cores were removed**,** and thus**,** the hollow carbonaceous structures formed. The resulting samples were rinsed with distilled water **three** times for further characterization.

### Characterization

The formation, growth, and etching processes of the as-prepared Au@C and Ag@C carbonaceous nanostructures were characterized using various techniques, including transmission electron microscopy (TEM, JEOL-1400), scanning electron microscopy (SEM, FEI Nova NanoSEM 230 FESEM), high-resolution TEM (HRTEM) using a Phillips CM200 field emission gun TEM operated at 200 kV, UV-Vis spectra by Cary 5000 UV-Vis NIR spectrophotometer with a 1-cm quartz cell, Fourier transform infrared (FT-IR) spectrum by Perkin Elmer Spotlight 400 FT-IR microscope (650–4000 cm^−1^), Raman spectroscopy (Renishaw RM1000 Raman spectrometer, excitation wavelength 514 nm), and a Philips X’pert Multipurpose X-ray Diffraction System using Cu-K_α_ (*λ* = 0.15406 nm) radiation at 40 kV and 100 mA, in the measurement range of 10–80° with a scanning step of 0.02°/s.

### Photothermal Measurements

A thermocouple was used for the measurement of photothermal temperature based on previous reports [23–25, 29–31]. The thermocouple measurement used Delta OHM HD2128.2 T-type. The light source is a laser power with a density of 0.17 W/cm^2^. To be more accurate and reliable, temperatures were measured by a Cedip Titanium 560 M IR camera, with 640 × 512-pixel resolution images at frame rates up to 100 Hz. The pixels are square with dimension 24 × 24 μm. The charge-coupled device (CCD) chip in the camera is sensitive to a wavelength of 3.6–5.1 μm, so only the thermal emission from the fluid can be seen in the images as the 0.8-μm laser wavelength is invisible to the camera’s sensor. The temperature range is set to a range of 0–60 °C. For the photothermal measurement, we have chosen the Au@C carbonaceous structures (Au nanorods with an aspect ratio of ~ 3.7), as a case study, because of the strong stability and non-toxicity of Au in biosystems.

## Results and Discussion

### Microstructure of Carbonaceous Ag@C Nanostructures

**Fig. 1:**
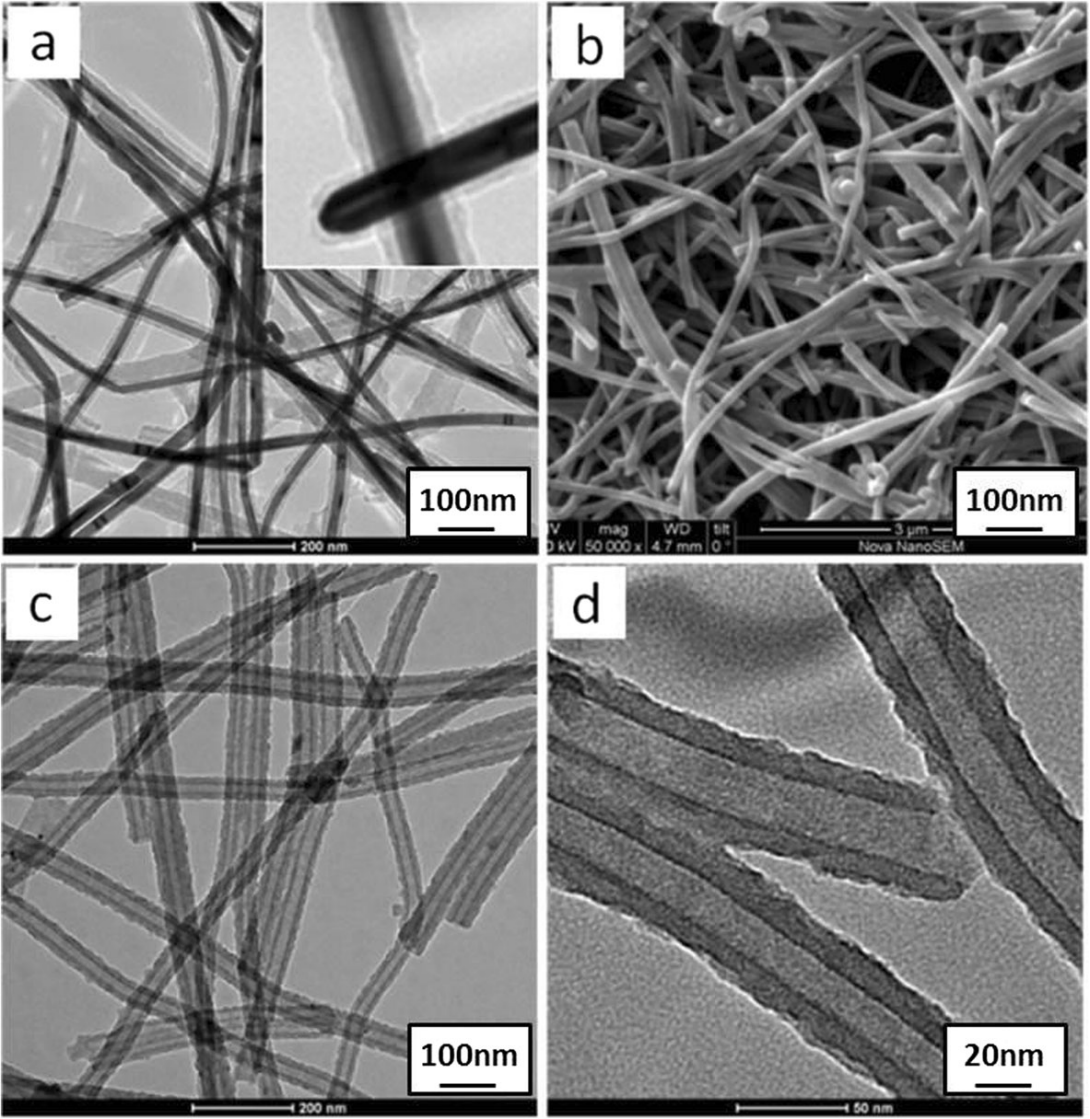
Ag@C nanostructures: **a** TEM and **b** SEM images for carbonaceous nanocables. **c**, **d** TEM for carbonaceous nanotubes

The morphology and composition of the achieved nanoparticles were characterized using TEM and SEM techniques. Figure 1a shows the TEM images of the as-prepared Ag@C nanocables, along with a magnification image inset, in which the diameter of cables was estimated as 30–50 nm while the Ag core of 10–20 nm in width. This is also confirmed by SEM, as shown in Fig. 1b, where the Ag@C nanocables are not straight but bended. Here, it was found that only one-dimensional Ag@C nanocables but carbonaceous spheres were formed, indicating that the self-nucleation and growth process via the carbonization of glucose have been significantly suppressed, and the coating process on Ag core is dominant under our reported conditions.

Subsequently, the Ag@C nanocables could be etched by keeping the same reaction solution at 60 °C for 24 h, while without the addition of etching agents, and then the hollow carbonaceous tubes were obtained, as shown in Fig. 1c, d with different magnifications. Clearly, the Ag core could be removed or etched from the nanocables (Fig. 1a) by the designed hydrothermal reaction. In comparison, the nanotubes are slightly shrunk but no damaged or broken pieces, suggesting that the carbonaceous shells are relatively stable in structure under the reported conditions.

**Fig. 2:**
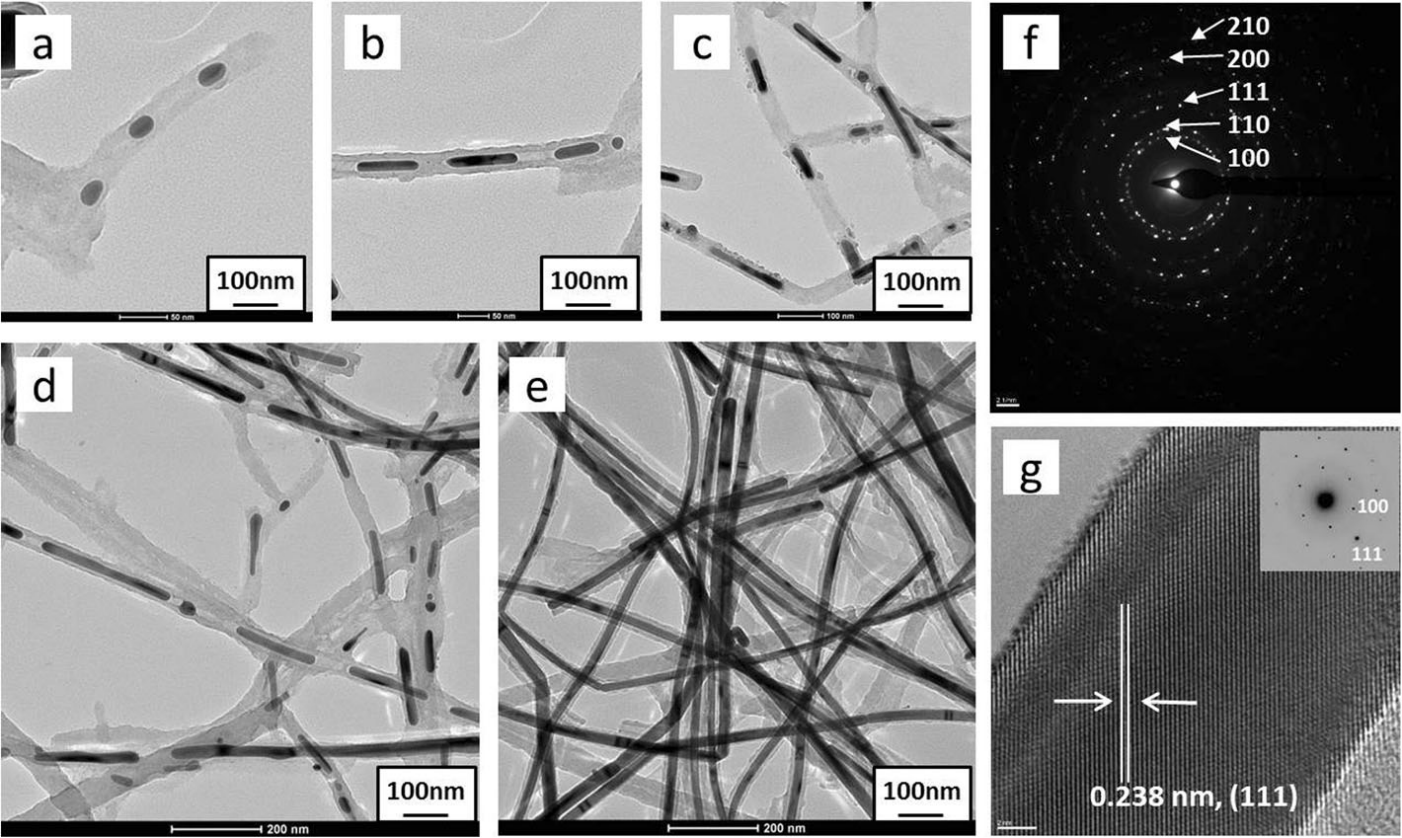
Ag@C core-shell structural formation process with time: **a** 1 h, **b** 3 h, **c** 6 h, **d** 12 h, and **e** 24 h; **f** ED pattern with indexed planes and **g** HRTEM of Ag rods of Ag@C nanocomposites with lattice space of 0.238 nm attributed to {111} planes

To better understand the formation and growth processes of Ag@C nanocomposites, different reaction times were taken to track the dynamic reaction (Fig. 2). With time increasing from 1 h (a) to 3 h (b), 6 h (c), 12 h (d), and 24 h (e) under the reaction temperature of 180 °C, the carbonaceous Ag@C was formed, in which the Ag gradually formed from dots (a) to short rods (b–d), and finally to rods/wires (e). The silver nanorods in the Ag@C nanostructures are well crystallized, confirmed by HRTEM images in Fig. 2f and g, along with electronic diffraction pattern inset of Fig. 2g. The crystalline Ag{111} planes with a lattice space of 0.238 nm were clearly inspected by HRTEM image (Fig. 2g).

According to current experiments, two possible mechanisms could be used for the explanation of the Ag@C formation. First, AgBr precipitate was formed by Br^−^ ions (from CTAB) reacting with Ag^+^ ions (from AgNO_3_) due to very small *K*_sp_ (5.0 × 10^−13^) at room temperature in aqueous solution, while AgBr is not thermally stable and thus form metallic Ag under the heat treatment, acting as a nucleation center for formation and growth of carbonaceous shell. The CTAB also played another role in surface modification, promoting the orientation formation of silver nanorods or nanowires, because the CTAB molecules are preferentially adsorbed on the long-axis crystal planes of the growing Ag particles [32]. Second, the glucose acted as a reducing agent to reduce free Ag^+^ ions to Ag^0^ atoms at a high temperature (> 140 °C) [33], because of its rich −OH and aldehyde groups. Then, the newly formed Ag nuclei acted as a nucleation center for glucose molecules adsorption and subsequently polymerized as a carbonaceous sheath on the silver backbone [34–37].

**Fig. 3:**
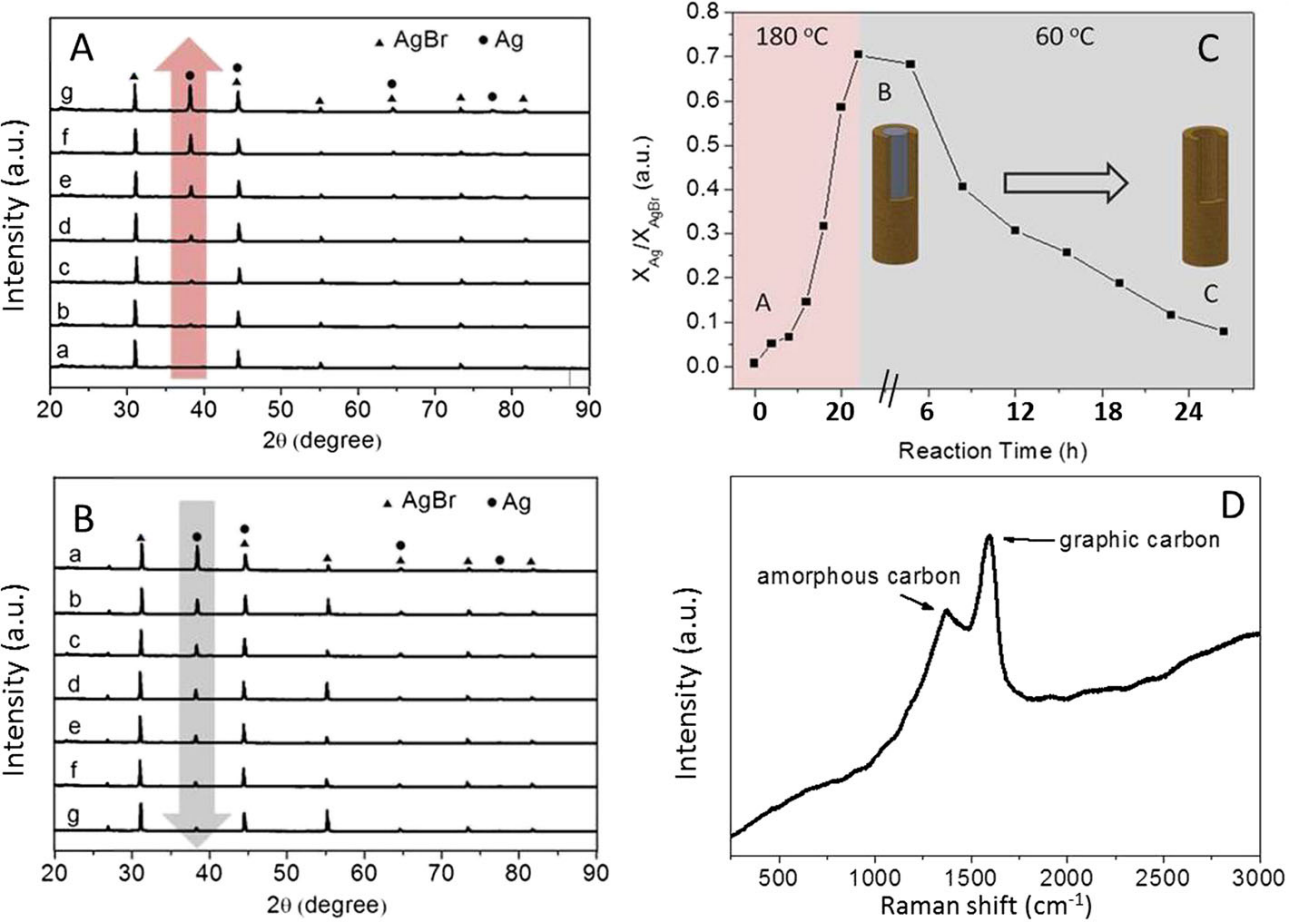
XRD illustrating the formation processes of Ag nanorods with carbonaceous shell (**A**) and Ag gradual self-digestion (**B**), the formation and etching/evolution curve for Ag core (**C**), and Raman spectrum for carbonaceous Ag@C nanocables (**D**)

As a further confirmation, the XRD technique was used to track the formation of Ag@C nanocables and carbonaceous sheath. Time-dependent XRD measurements were employed to monitor the composition change of the intermediates and final products of Ag@C nanoparticles. The overall process consists of two stages depending on the reaction temperature. At the first stage, glucose, AgNO_3_, and CTAB were used as the starting materials for the preparation of Ag@C nanocables at a high temperature (180 °C). In the second stage, the reaction temperature of the same system was decreased to 60 °C for hydrothermal treatment. Time intervals are 4 and 3 h for the growth and etching processes, respectively, as shown in Fig. 3c.

Figure 3 shows the XRD patterns that the initially formed brown color precipitate and displays two intensive diffraction peaks, indexed as cubic AgBr (200) and (220) (JCPDS card no. 079-0149), respectively. In the beginning, no Ag diffraction peaks were observed until the reaction was proceeded over 8 h under heating, in which the metallic Ag(111) peak emerged at ~ 38.1° (JCPDS card no. 087-0717). A gradual increase in the intensity of the typical Ag(111) peak with time indicates that the metallic Ag formed and reached its maximum intensity around 24 h under the reported conditions. It was noted that some diffraction peaks, e.g., Ag(200) and AgBr(220), are overlapped in the XRD pattern somehow. Interestingly, when the reaction temperature was adjusted down to 60 °C, the etching process of Ag cores occurred, confirmed by the gradually reduced intensity of the typical Ag(111) diffraction peak located at ~ 38.1°. Over tens of hours, the etching resulted in the disappearance of Ag cores, confirmed by the nearly disappeared Ag(111) peak. The analysis of product as a function of reaction time has been conducted, as shown in Fig. 3c. This indicates the Ag cores could be almost removed under the thermal conditions, consistent with the TEM observations (Fig. 1c, d). In addition, Raman spectrum (Fig. 3D) shows the formation of both types of carbon: amorphous and graphic in this reaction system.

Based on the above XRD analysis, a thermodynamic equilibrium may exist between the reduction of Ag^+^ ions by glucose to form Ag cores and the oxidation of Ag by Br^−^ ions, consistent with previous reports [38–40]. For example, Zhou et al. [39] demonstrated that Ag nanowires were not formed until the reaction temperature was above 140 °C, confirmed by Hussain’s work [40]. For the carbonization of carbohydrates (e.g., glucose, saccharide), it was widely accepted that the carbonaceous product can be synthesized at the temperature ranging from 170 to 240 °C [41, 42]. However, the carbonaceous sheath of the nanocables is amorphous, different from those carbon nanotubes or graphene obtained under high-temperature annealing treatments [1–3].

**Fig. 4:**
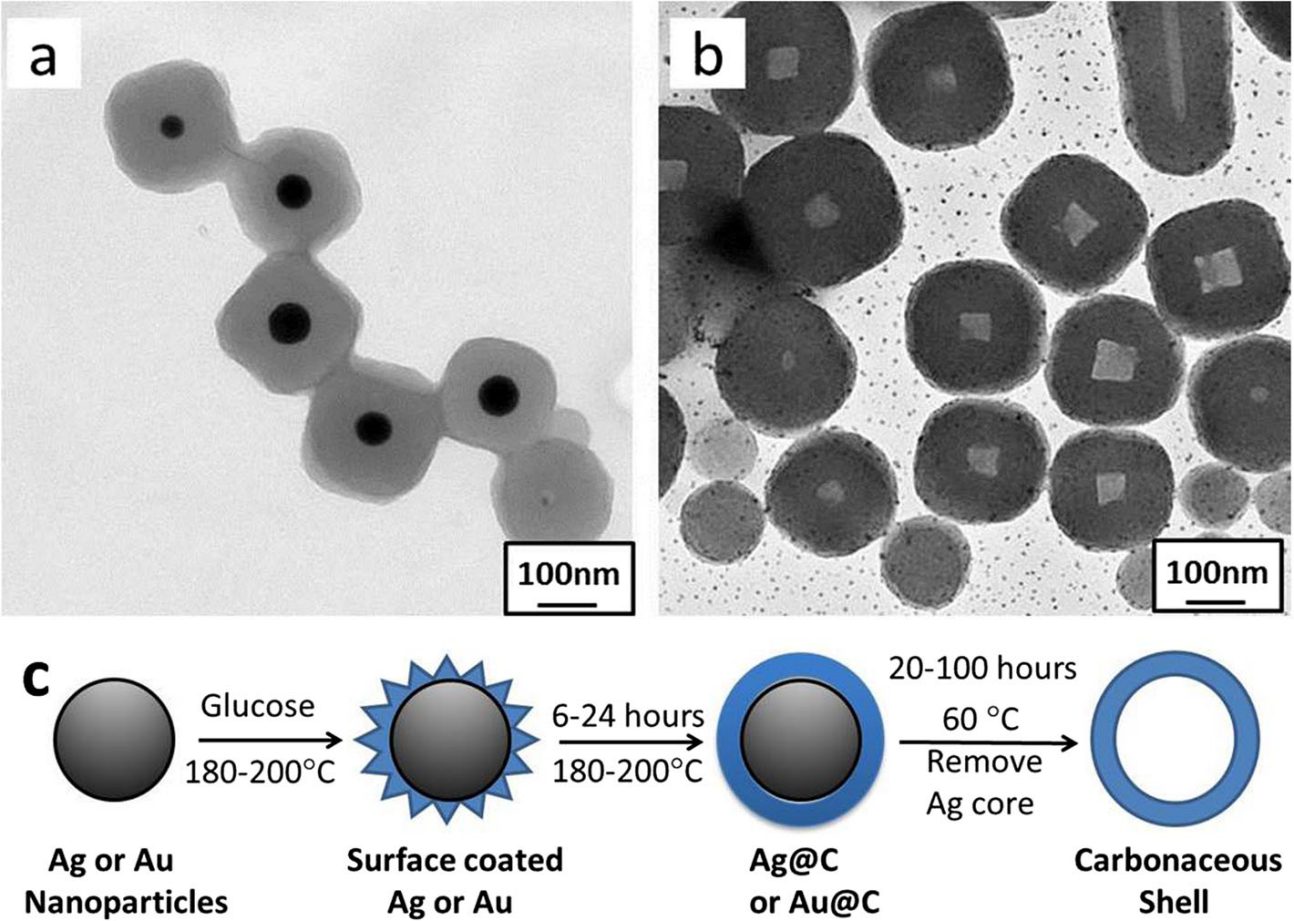
The Ag core etching from Ag@C core-shell nanoparticles (**a**) to hollow carbonaceous structure (**b**). **c** A diagrammatic scheme illustrating the formation and self-digestion of Ag@C nanostructures

Specifically in etching Ag cores, the oxygen molecules possess a high electrode potential of *E*_0_ = +1.229 V vs. standard hydrogen electrode (SHE), which is sufficient to oxidize Ag^0^ to Ag^+^ ions in the considered system. This can be further supported by previous studies, in which the oxidized etching in the presence of halide ions (Br^−^) to the metal nanoparticles with regular shapes is anisotropic in all directions [43, 44]. Xu et al. [43] reported that the Cl^−^ or Br^−^ ions preferred to etch (110) plane of Ag nanocrystals. This selectivity may be attributed to the difference in surface free energies of Ag crystal planes (111, 110, and 100). Guo et al. [44] controlled the selective etching starting point from the nanorod side by reducing surface passivation of surfactant and hence increasing reactivity on the side planes. The Ag core etching was also confirmed by our experimental observations for the Ag@C nanoparticles under similar reaction conditions, as shown in Fig. 4. The small black dots (Fig. 4b) may be originated from the decomposition of AgBr as Ag dots under a thermal treatment. A diagrammatic scheme can be used to illustrate the synthesis process of Ag@C and the formation of hollow carbonaceous shell (Fig. 4c).

**Fig. 5:**
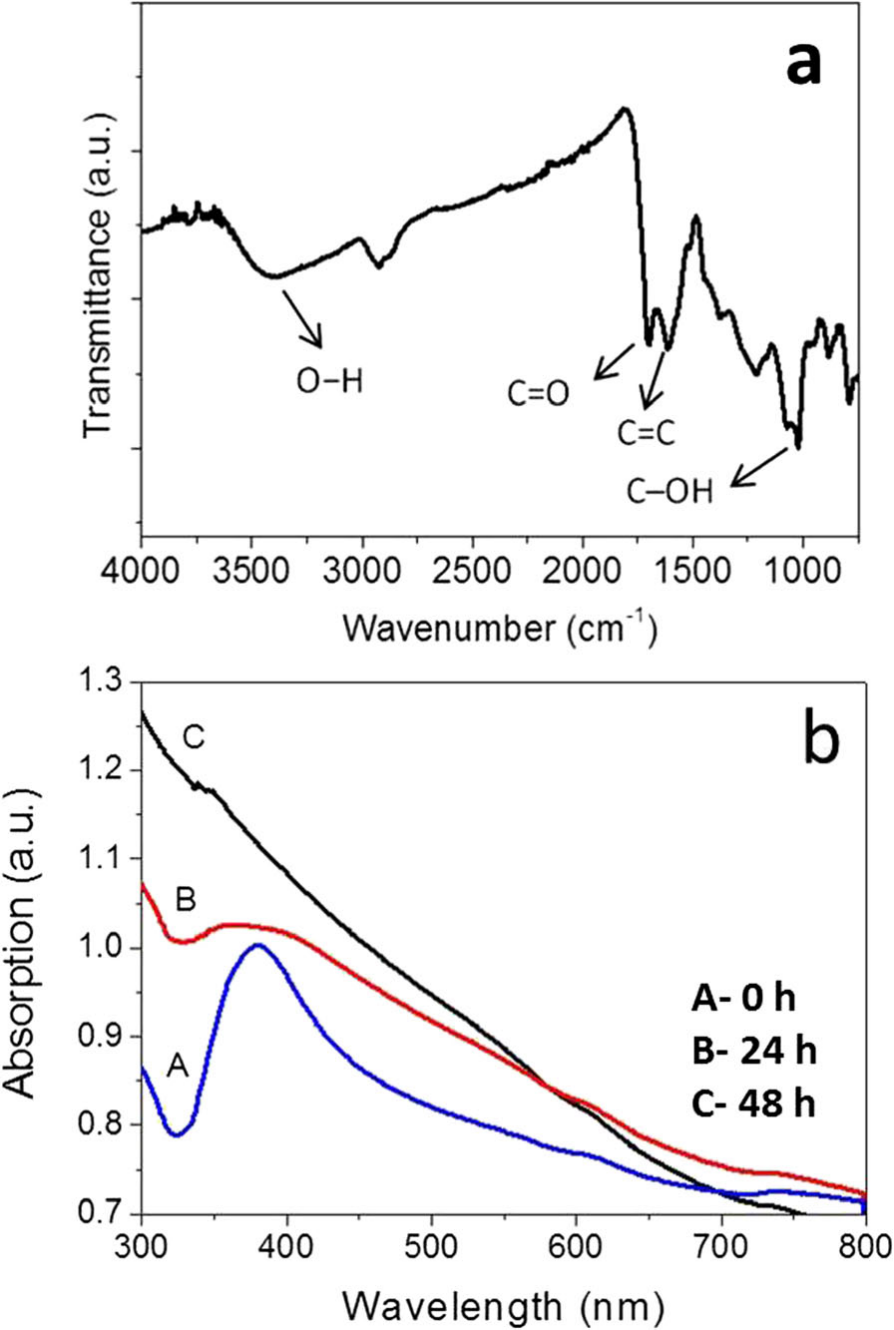
The optical properties of Ag@C nanocomposites and hollow carbonaceous structures measured by FT-IR spectrum (**a**) and UV-Visible spectra (**b**)

The corresponding UV-Vis property of the as-synthesized Ag@C core-shell nanocables and hollow carbonaceous structure were also measured, as shown in Fig. 5. This can also further confirm that the Ag core was etched from nanocables. The Ag@C nanocables show an intensive absorption peak located at ~ 382 nm (curve A in Fig. 5b), indicating the existence of metallic silver, which can produce strong surface plasma resonance [39]. On the contrary, the carbonaceous nanotubes show no absorption peaks, as shown in the curve C of Fig. 5b. In addition, the FT-IR spectrum (Fig. 5a) is mainly to confirm the formation of carbonaceous sheath, rich in functional groups such as C=O, C–OH, and OH [35].

The availability of this synthesis method was validated to be a general one, not only for silver but also for other noble metals (e.g., gold). Here, the AuCl_4_^−^ ions were selected, and they are easier to be reduced by glucose than Ag^+^ ions under the same conditions, since the standard electrochemical potential of AuCl_4_^−^/Au^0^ pair (0.99 V vs. SHE) is higher than that of Ag^+^/Ag^0^ pair (0.799 V vs. SHE) [45, 46]. Importantly, Au is more stable than Ag and not toxic, which is beneficial for photothermal effect in biomedical applications.

**Fig. 6:**
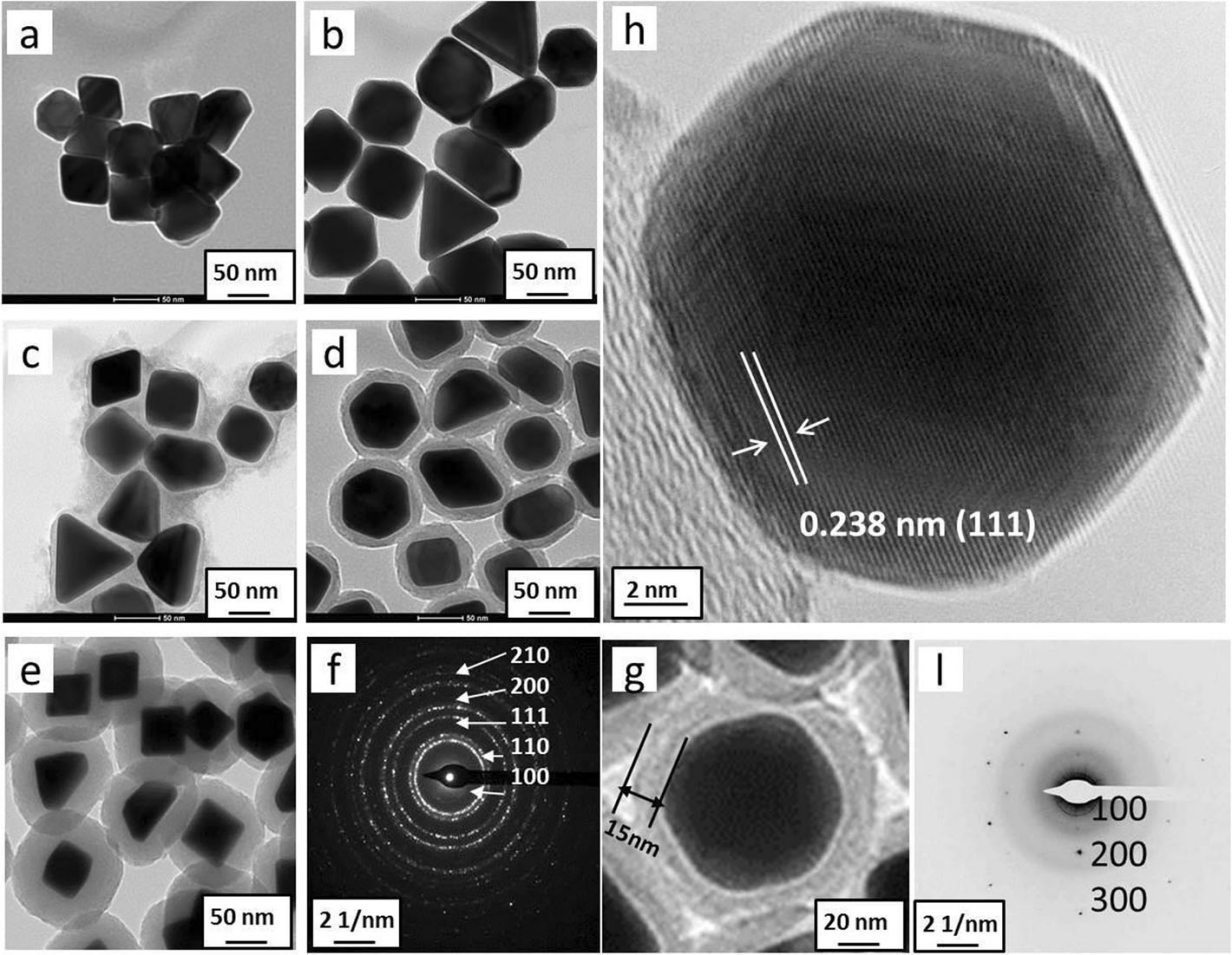
Au@C core-shell structure formed with time: **a** 1 h, **b** 3 h, **c** 6 h, **d** 12 h, and **e** 24 h, respectively; **f** ED pattern for particles and the diffraction rings were indexed as (100), (110), (111), (200), and (210); **g** a single Au@C nanoparticle, along with its HRTEM image (**h**) and the indexed ED pattern (**i**) showing in a single crystal

Figure 6 shows the Au@C carbonaceous nanostructures were formed with time. It was clearly observed that the non-spherical Au nanoparticles (Fig. 6a) were formed within 1 h under the reported conditions, where the AuCl_4_^−^ ions were reduced by glucose first; meanwhile, the carbonaceous sheath was formed surrounding Au nanoparticles via polymerization of glucose at a temperature of 180 °C for a couple of hours, as shown in Fig. 6c–e. With time increasing, the carbonaceous sheath thickness became thicker from a few nanometers (Fig. 6d) to tens of nanometers (Fig. 6e), and the sheath geometry is highly related to the shape of the Au particles themselves. The electronic diffraction pattern of Ag@C core-shell nanoparticles was indexed as diffraction rings, corresponding to (100), (110), (111), (200), and (210) planes (Fig. 6f), respectively.

The crystalline lattice of single Au nanocrystal (Fig. 6g) in the core-shell structure was characterized by the HRTEM technique. Figure 6h shows the HRTEM image that the lattice spacing of ~ 0.238 nm could be assigned to Au(111) plane [47, 48]. The electronic diffraction spots in Fig. 6i were indexed as (100), (200), and (300) planes, suggesting the formed single Au crystal under the reported conditions.

**Fig. 7:**
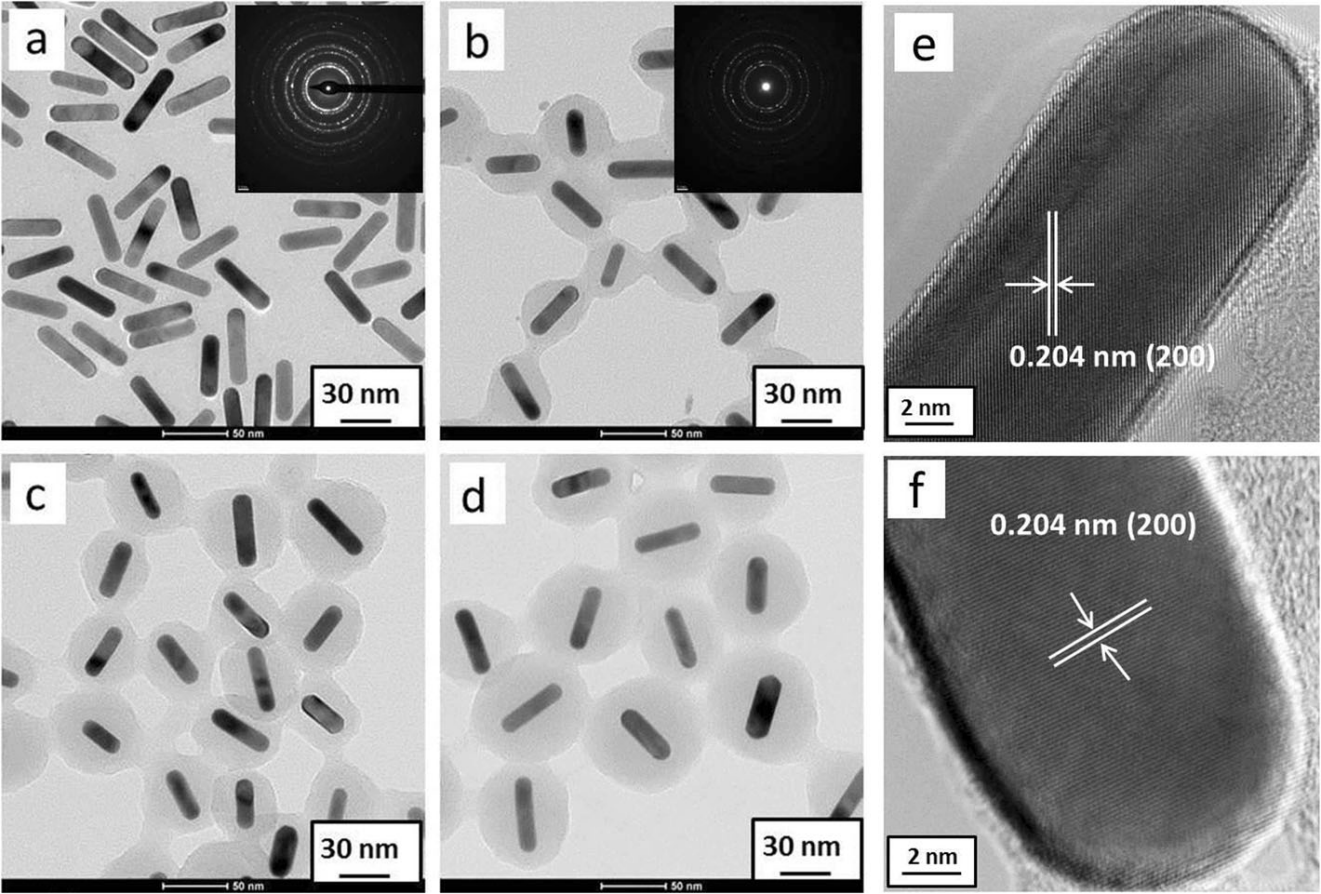
Au@C core-shell structure obtained with different reaction time: **a** 0 h (original gold nanorods), **b** 4 h, **c** 12 h, and **d** 24 h. **e** HRTEM image for a single Au rod. **f** HRTEM image for a single Au@C carbonaceous nanorod

To further confirm the proposed synthesis strategy, Au nanorods were also available for carbonaceous coating as core-shell structures under the reported hydrothermal conditions. Figure 7 shows the coating process of carbonaceous sheath on Au nanorods with aspect ratio of ~ 3.7, as a case study. With time increasing, the thickness of carbonaceous sheath on Au nanorods became thicker from ~ 6 nm (Fig. 7b) to ~ 15 nm (Fig. 7c) and ~ 23 nm (Fig. 7d), and the carbonaceous sheath grows to be sphere-like after 24 h, not affected by the length of Au nanorods (Fig. 7d). A significant contrast for carbonaceous sheath (grey color) and gold nanorods (black color) was observed. Figure 7e and f show the HRTEM images of single Au nanorod before and after carbonaceous coating. The lattice spacing of ~ 0.204 nm could be assigned to Au(200) plane. The energy dispersion spectroscopic (EDS) analysis for Au@C and Ag@C nanostructures have been conducted, as shown in Additional file 1: Figure S1. It was found that the carbonaceous sheath has less impact on the structure of gold single crystals in this work.

### Optical Properties

**Fig. 8:**
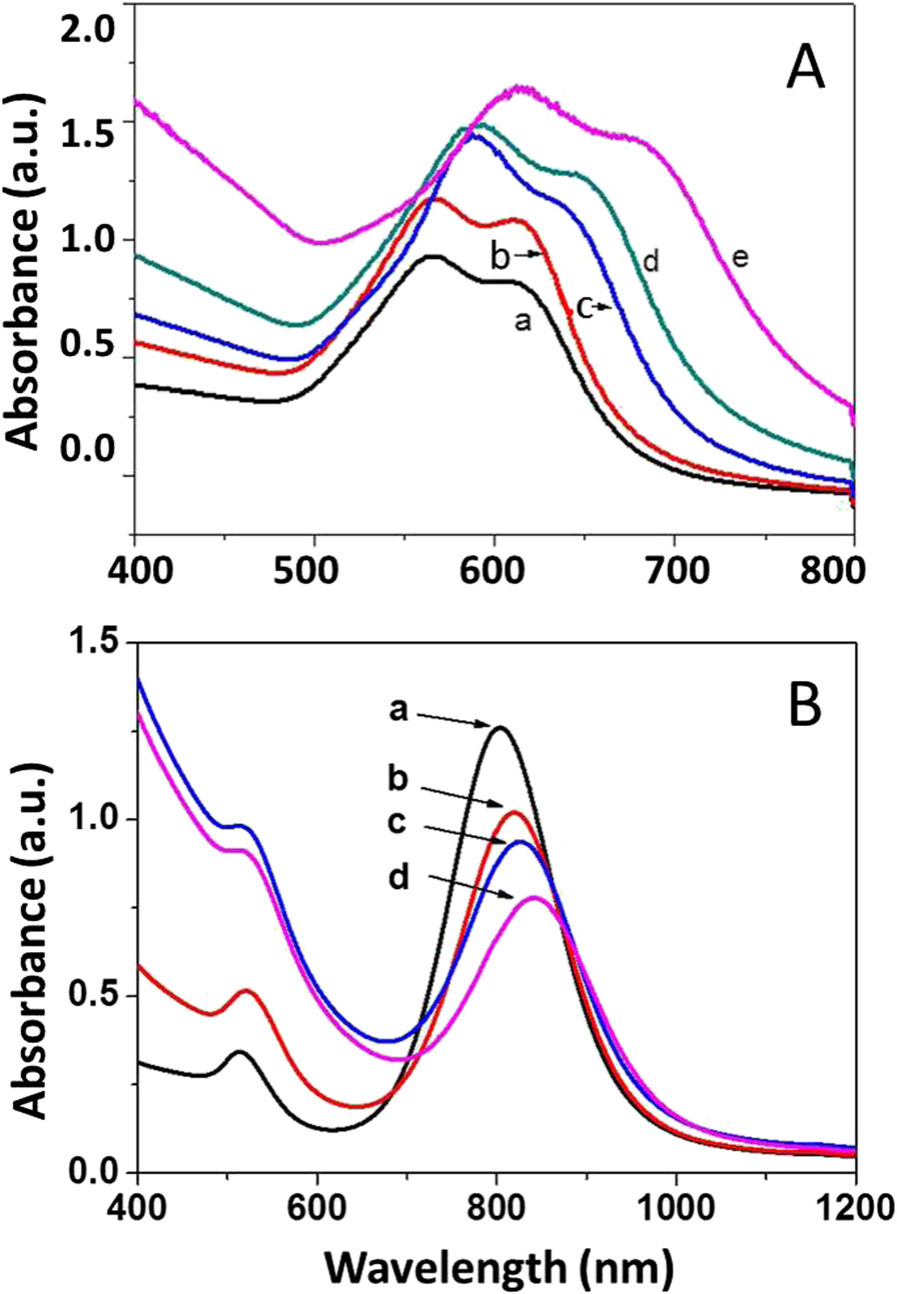
UV-Vis spectra for Au@C nanocomposites: **A** Au@C carbonaceous non-spherical nanoparticles obtained at 1, 3, 6, 12, and 24 h, respectively. **B** Au@C carbonaceous nanorods obtained at 0, 4, 12, and 24 h, respectively

The corresponding optical properties of Au@C nanostructures were measured by UV-Vis spectroscopy, as shown in Fig. 8. The UV-Vis absorption spectra for Au@C nanoparticles (Fig. 6a–e) red-shift gradually from 560 nm (a) to ~ 565 nm (b), ~ 580 nm (c), ~ 590 nm (d), and ~ 620 nm (e), respectively, for the intense absorption peak in Fig. 8A, with sheath thickness increasing (Fig. 6c–d). Similarly, for the Au@C nanorods in Fig. 7a–d, the thickness of carbonaceous sheath influences the intense surface plasmon resonance (longitudinal mode) of Au nanorods and leads to red shifting from original ~ 810 nm (a) to ~ 820 nm (b), ~ 826 nm (c), and ~ 848 nm (d), respectively, as shown in Fig. 8B, while another surface plasmon resonance (transverse mode) located at around 518 nm is nearly kept the same position. That is, the carbonaceous sheath indeed affects the surface plasmon resonance of the Au@C nanocomposites [36].

As well known, Au is a noble metal and its nanoparticles can show intense surface plasmon resonance (SPR), which can be influenced by its size, morphology, and surrounding media. Both the Au@C non-spherical nanoparticles (Fig. 6a–e) and the Au@C nanorods (Fig. 7a–d) show intense SPR with gradually red-shifting peaks in the UV-Vis spectra (Fig. 8A, B), probably caused by the thickness increase of carbonaceous shell on Au surface. This can be supported by the previous studies [49–52]. The authors reported that the surface modification of photonic nanostructures such as gold (Au) nanoparticles can lead to novel physical phenomena including selective light-matter interactions and rapid energy transfer processes [49–51]. Others reported that the carbon component can also influence the light-matter interactions for such hybrid systems, for example, by reducing scattering effects. The presence of multilayer graphene shell (sub-5 nm thickness) influenced the optical properties and stability (chemical and thermal) of the encapsulated Au nanoparticles [52].

**Fig. 9:**
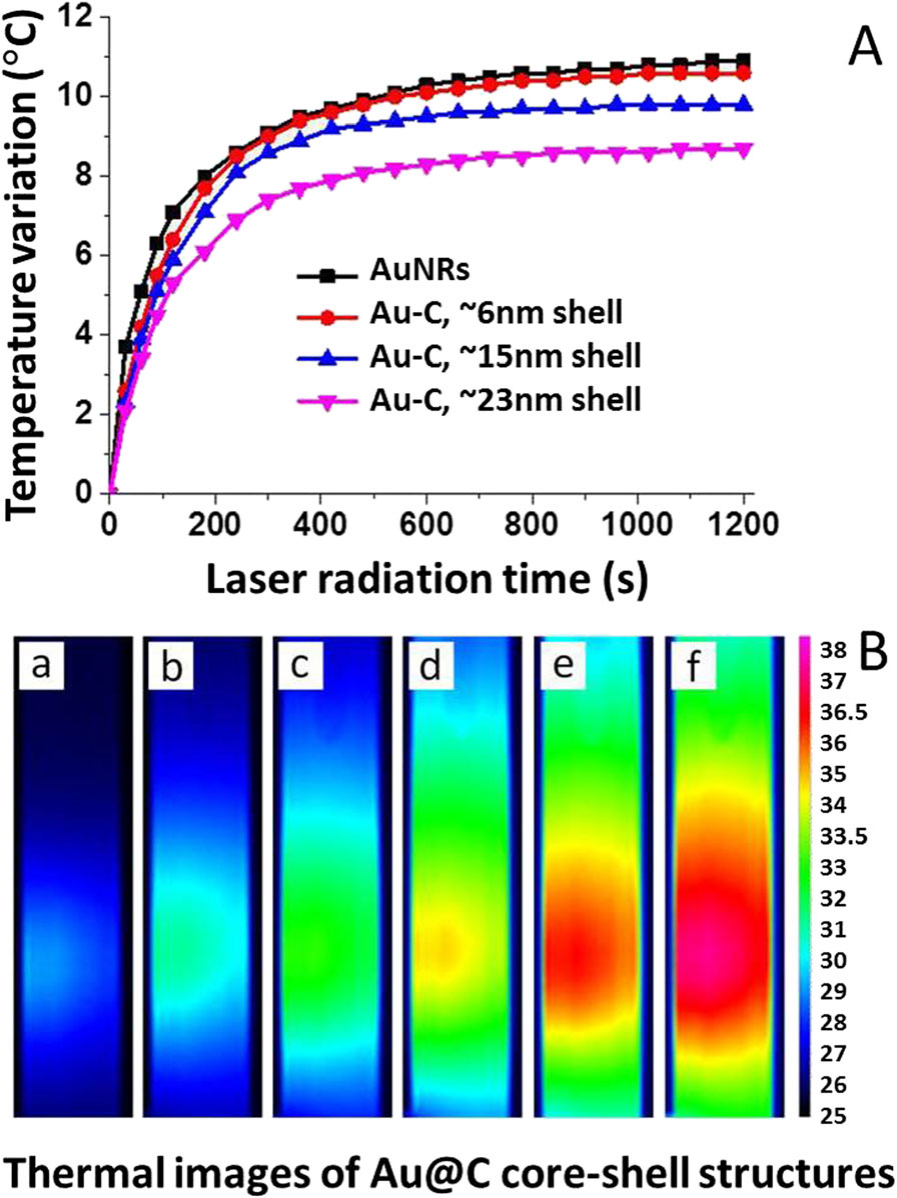
Laser-induced test of photothermal effect of Au@C carbonaceous nanocomposite with laser power density of 0.17 W/cm^2^. **A** The relationship between temperature and irradiation time for the aqueous solution of Au@C nanostructures with different thicknesses: bare Au nanorods with no carbonaceous coating, ~ 6 nm, ~ 15 nm, and ~ 23 nm, in which the concentration of each system was adjusted to give an extinction intensity of 1.0 at 800 nm. **B** Photothermal images of Au@C carbonaceous nanostructures represent different times at (a) 30, (b) 60, (c) 90, (d)180, (e) 360 and (f) 840 seconds under 0.17 W/cm^−2^ irradiation

The laser-induced thermal (photothermal) property of the Au@C carbonaceous nanostructures was examined. Au@C was chosen as a case study, because Au is more stable than Ag and is non-toxic in biosystems. Figure 9 shows the photothermal property of Au@C with different shell thicknesses of around 6 nm, 15 nm, and 23 nm. The color change from blue (cold) to red (hot) which indicates temperature rising within the Au@C suspension. The detailed temperature changes were recorded and shown in Fig. 9B. Within the first 600-s irradiation, the temperature rise is rapid for all the four samples. The temperature asymptotically approached a steady-state over 1200 s. Clearly, the surface coating may reduce light absorption for Au@C nanocomposites, exhibiting lower temperature than bare Au nanorods as ~ 10.6 °C to ~ 9.3 °C and ~ 8.1 °C, respectively, corresponding to ~ 6 nm, ~ 15 nm, and ~ 23 nm carbonaceous shell coated Au nanorods. A similar trend was observed for Au@SiO_2_ nanocomposites, where a thicker SiO_2_ shell led to a decrease in temperature, e.g., a 5-nm SiO_2_ shell resulted in lowering ~ 5 °C as compared to the bare Au nanorods [53].

Although the carbonaceous shell led to temperature decreasing, it is still attractive because the bare Au nanoparticles are not available for direct applications in biosystems due to the reaction with biomolecules (proteins, amines). Of course, the competing parameters should be optimized for a particular application in future work. In general, the carbon shell should be well controlled so as not to make it too thick to avoid a significant reduction in light absorption, and also the shell is not too thin to be broken under light irradiation. Obviously, both the bare Au nanorods and the Au@C nanocomposites can lead a significant temperature rise to a proper range (e.g., 37–47 °C) by laser irradiation, potential for photothermal therapy, while associated with targeted drug molecules. Regarding the relationship between optical and photothermal properties, both of them for Au@C nanostructures are dependent on SPR from noble metallic Au. The SPR, a unique phenomenon to plasmonic Au nanoparticles, leads to strong electromagnetic fields on the particle surface and consequently enhances all the radiative properties such as absorption and scattering. Additionally, the strongly absorbed light is converted to heat quickly via a series of nonradiative processes, if external light acts on the surface of Au nanoparticles. As noted, the photothermal property is mainly caused by NIR light but not visible light [54]. It is needed to be pointed out that more works are needed to be conducted in the near future, focusing on the optimization of pertinent parameters in concentration, pH, particle size, and surface modification for real biomedical applications of such Au@C carbonaceous nanocomposites.

## Conclusions

This study developed a general strategy for the synthesis of noble metal carbonaceous nanocomposities (Au@C, Ag@C) with different morphologies by controlling hydrothermal conditions. A few interesting findings can be summarized as follows:

i) A high-temperature hydrothermal reaction (180–200 °C) could result in the formation of noble metal@C core-shell nanostructures, in which the glucose played a multiple role: reductant for metallic ions, shape modifier, and surface protection;
ii) A low-temperature (60–100 °C) hydrothermal reaction for the Ag@C core-shell system was beneficial for etching Ag to obtain hollow carbonaceous sheath, due to the etchant (Br^−^/O_2_). This may open a simple path for fabricating hollow carbon nanostructures (e.g., tubes). However, the Au@C core-shell nanoparticles are quite stable under the same conditions;
iii) The generated Ag or Au cores are well crystallized, but the carbonaceous sheath is amorphous; and
iv) The rodlike Au@C nanocomposites were laser-induced to show photothermal effect, and the thickness of carbonaceous shell can tune the photothermal temperature, potential for biomedical applications.

Generally, this work may offer a simple but effective synthesis strategy, not only for noble metals but also for other metals, metal oxides, and inorganic materials to design and construct controllable carbonaceous nanostructures with potential applications in sensor, energy storage, catalysis, and biomedicine.

## Supplementary information

**Additional file 1.** Supporting Information.

## Data Availability

The data and material provided in this study are available in Additional file 1.

## Abbreviations

Ag@C: Silver carbonaceous nanostructure
Au@C: Gold carbonaceous nanostructure
CCD: Charge-coupled device
CTAB: Cetyltrimethylammonium bromide
EDS: Energy dispersion spectroscopy
FT-IR: Fourier transform infrared
GO: Graphene oxide
HRTEM: High-resolution transmission electron microscopy
PVA: Poly(vinyl alcohol)
SEM: Scanning electron microscopy
SHE: Standard hydrogen electrode
SPR: Surface plasmon resonance
TEM: Transmission electron microscopy
UV-Vis: Ultraviolet-visible spectrometer
XRD: X-ray diffraction
