# Supplementary material for: Development of a General Fabrication Strategy for Carbonaceous Noble Metal Nanocomposites with Photothermal Property
Source: Nanoscale Res Lett. 2020 Jan 21;15:17. doi: 10.1186/s11671-019-3242-1 (PMC6974232; doi:10.1186/s11671-019-3242-1)

Supporting Information

Development of aGeneral Fabrication Strategy for CarbonaceousNoble MetalNanocompositeswith Photothermal Property

HongmeiZhu,[a] Xuchuan Jiang[b,*]

[a]*School of Mechanical Engineering, University of South China, Hengyang421001, Hunan, China*

[b]*Department of Chemical Engineering, Monash University, Clayton, VIC 3800, Australia*

*Email: Xuchuan.jiang@monash.edu

The energy dispersion spectroscopic (EDS) analysis for Au@C and Ag@C nanostructures have been conducted, as shown in Fig. S1.

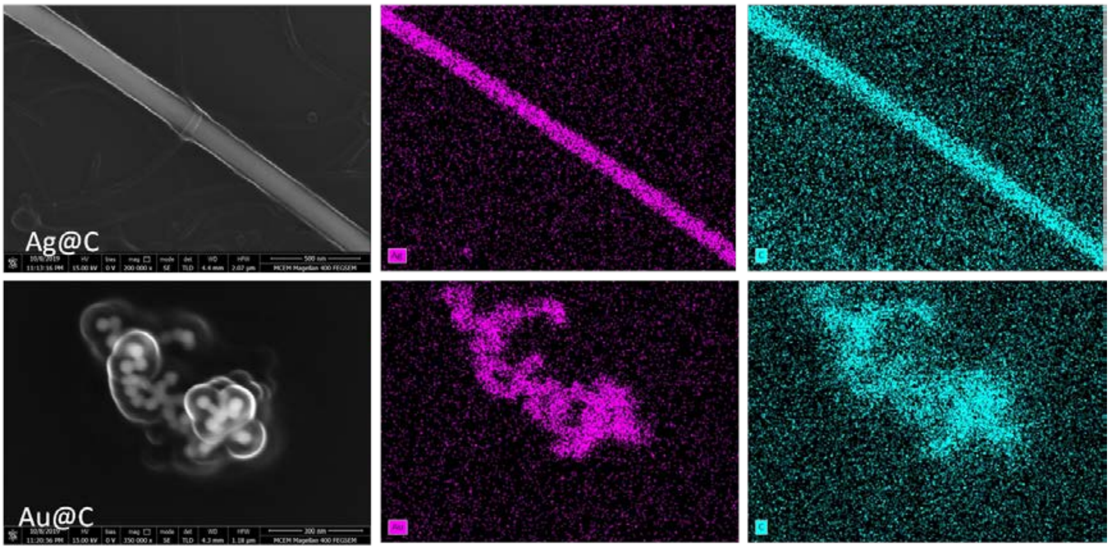

Supplement: Supplementary file 1 — Additional file 1. Supporting Information. [file 11671_2019_3242_MOESM1_ESM.pdf]
